# Exploring the Virome of *Gyropsylla spegazziniana*: A Major Yerba Mate Pest

**DOI:** 10.3390/pathogens15060620

**Published:** 2026-06-09

**Authors:** Yesica Gisel Candia, Vanesa Nahirñak, Alejandra Badaracco, Humberto Debat, María Elena Schapovaloff, Nicolás Bejerman

**Affiliations:** 1 Consejo Nacional de Investigaciones Científicas y Técnicas (CONICET), Buenos Aires CP1033, Argentina; candia.gisel@inta.gob.ar (Y.G.C.); nahirnak.vanesa@inta.gob.ar (V.N.); badaracco.alejandra@inta.gob.ar (A.B.); debat.humberto@inta.gob.ar (H.D.); 2 Estación Experimental Agropecuaria Montecarlo, Instituto Nacional de Tecnología Agropecuaria (INTA), Buenos Aires CP3384, Argentina; 3 Instituto de Patologia Vegetal (IPAVE)–Instituto Nacional de Tecnología Agropecuaria (INTA), Córdoba CP5019, Argentina

**Keywords:** *Gyropsylla spegazziniana*, virome, yerba mate pest, RNA viruses

## Abstract

The yerba mate psyllid, *Gyropsylla spegazziniana*, represents a major pest affecting yerba mate production, leading to considerable economic losses. Although several aspects of its ecology and management have been investigated, little is known about the viruses associated with this insect pest. In this study, we conducted the first RNA high-throughput sequencing (HTS) analysis of *G. spegazziniana* to examine its virome, uncovering a diverse array of previously undescribed RNA viruses. Our analysis led to the identification of five novel viruses spanning different viral lineages. These include representatives with evolutionary affinities to beny-like, picorna-like, and sobemo-like viruses, provisionally designated as *Gyropsylla spegazziniana* beny-like virus 1 (GSBlV1), Gyropsylla spegazziniana picorna-like virus 1 (GSPlV1), and Gyropsylla spegazziniana sobemo-like virus 1-3 (GSSlV1-3), respectively. Phylogenetic analysis of the bi-segmented, highly divergent sobemo-like viruses revealed that these viruses are grouped with other insect-associated sobemo-like viruses. The beny-like virus clustered together with other insect-associated beny-like viruses, whereas the picorna-like virus clustered together with psyllid-associated picorna-like viruses. Overall, these findings demonstrate that *G. spegazziniana* harbors a complex and previously uncharacterized virome. This work provides a foundation for further research into the ecological significance, evolutionary patterns, and possible use of these viruses in biological control strategies of this major pest within yerba mate agroecosystems.

## 1. Introduction

The superfamily *Psylloidea* (psyllids) represents an ecologically diverse but relatively understudied group within the suborder *Sternorrhyncha*, order *Hemiptera* [1,2]. Members of this group, commonly referred to as psyllids or jumping plant lice, are sap-sucking insects highly specialized in their host plants [1,2]. Some psyllid species are among the most devastating agricultural pests worldwide, either due to their ability to transmit plant pathogens [3] or the range of phenotypes they induce in host plants through salivary secretions [2].

In Argentina, around 16% of the 470 psyllid species known in the Neotropical region have been reported, and several of them are considered economically important pests [4]. *Gyropsylla spegazziniana* (Lizer & Trelles), commonly known as the yerba mate psyllid or “rulo”, belongs to the family *Aphalaridae* [5], and is one of the major pests of yerba mate (*Ilex paraguariensis* A. St-Hil.). This crop holds substantial regional importance, as its leaves and stems are commonly used to prepare a traditional beverage known as ’mate’ widely consumed in Argentina, Paraguay, Uruguay, and Brazil [6]. *Gyropsylla spegazziniana* induces the formation of leaf deformations, referred to as “galls” or “rulos”, which serve as protective structures for its eggs and nymphs until adulthood, causing important economic losses in yerba mate production [7].

Although several studies have examined the biology, ecology, morphology, and control of *G. spegazziniana* [7,8,9,10,11], its virome remains uncharacterized. This lack of data hinders a comprehensive understanding of the role that viral communities play in shaping host population trends and their capacity to cope with environmental challenges. Because viral infections can deeply impact arthropod physiology, behavior, and tolerance to stressors [12], these host–virus interactions may strongly influence the emergence of pest outbreaks as well as the effectiveness of control measures [13]. Consequently, characterizing the viral community associated with *G. spegazziniana*, including its composition and diversity, is an essential step toward developing effective pest management strategies.

The landscape of viral discovery has been fundamentally transformed by the advent of high-throughput sequencing (HTS), which has enabled the systematic characterization of the “dark matter” within the global virosphere [14]. Unlike traditional culture-dependent or PCR-based methods that require prior knowledge of viral sequences, HTS offers an agnostic approach that allows for the detection of viruses in a host-neutral manner [15]. This technological shift has been particularly impactful in the study of invertebrates, where large-scale meta-transcriptomic studies have redefined the known virosphere by uncovering thousands of novel species and filling significant gaps in RNA virus phylogeny [16]. Recent expansive surveys have further extended this reach into aquatic environments, revealing a staggering and previously hidden diversity of RNA viruses within marine invertebrate ecosystems [17]. Furthermore, the sensitivity of HTS allows for high-resolution, in-depth assessments of the viromes associated with individual species, as demonstrated by the characterization of complex viral communities within the golden orb-weaver spider, Nephila clavipes [18]. Collectively, these advancements have reshaped our understanding of the breadth and evolution of the invertebrate virosphere.

In this work, we carried out the first high-throughput sequencing (HTS) analysis of *G. spegazziniana*, with the objective of exploring the virome of this major yerba mate pest. Our approach centered on uncovering the taxonomy and diversity of previously undescribed viral lineages, thereby bringing to light an overlooked component that may influence the biology of this psyllid. Through this virome exploration, we sought to generate a knowledge base that can support future research into the ecological functions of viruses associated with this agriculturally relevant species. Gaining insight into the viral community of *G. spegazziniana* also enhances our broader understanding of insect–virus relationships within agroecosystems, with implications for both ecological and evolutionary research. Such information may ultimately contribute to the design of innovative, virus-based pest control strategies. Overall, this study represents an important step toward elucidating a key but understudied aspect of this economically significant insect, with potential benefits for the sustainable management of yerba mate crops. Our findings include the discovery of five previously unknown RNA viruses, representing, to our knowledge, the first viruses reported in association with this major pest, demonstrating that the virome of *G. spegazziniana* is both rich and highly diverse.

## 2. Material and Methods

### 2.1. Psyllid Sampling and Sample Processing

Psyllids were collected in yerba mate plants located in Montecarlo, Misiones, Argentina and were used to establish colonies that were maintained at the EEA INTA-Montecarlo greenhouse facility on yerba mate plants under greenhouse conditions [5]. Adults were collected from the artificial rearing population of the psyllid for high-throughput analysis (HTS).

### 2.2. RNA Extraction and HTS Analysis

RNA was extracted from ten pooled psyllids. Insects were ground using a mortar and pestle in liquid nitrogen, and total RNA was extracted using the SV Total RNA Isolation System (Promega, Madison, WI, US) following the manufacturer’s instructions. Resulting RNAs were sent to Macrogen INC (Seoul, Republic of Korea) for library preparation and HTS. Library was prepared using the TruSeq Stranded Total RNA Library Prep Kit with Ribo-Zero Gold (Illumina, San Diego, CA, USA). Ribodepleted RNAs were then sequenced in paired-end 101 bp read format on an Illumina NovaSeq6000 platform.

The raw paired-end sequencing reads were first processed to remove low-quality regions and adapter sequences using *Trimmomatic* (v0.40; http://www.usadellab.org/cms/?page=trimmomatic, accessed on 11 January 2026). Default settings were applied with one key modification: the quality threshold was increased from 20 to 30, including adjustments during the initial ILLUMINACLIP step and the sliding-window trimming procedure, ensuring a minimum average quality score of 30. After filtering, the high-quality paired reads were assembled de novo with *rnaSPAdes* under standard conditions using the Galaxy platform (https://usegalaxy.org/, accessed on 11 January 2026). The assembled contigs were subsequently compared against a comprehensive non-redundant (NR) database of viral proteins obtained from NCBI (https://www.ncbi.nlm.nih.gov/protein/?term=txid10239[Organism:exp], accessed on 11 January 2026) using local BLASTX searches with an E-value cutoff of 1 × 10^−5^. All candidate viral hits identified in this step were carefully evaluated.

Contigs showing similarity to viral sequences were then subjected to further refinement. This included manual curation, extension, and validation through an iterative read-mapping approach. In this process, reads corresponding to each candidate contig were selectively extracted and used to extend the sequence; the updated contig was then re-used as a query in successive rounds until no further extension was possible. Finally, the resulting extended and curated transcripts were re-assembled using the alignment tool in *Geneious* (v8.1.9; Biomatters Ltd., Boston, MA, USA), applying high-sensitivity settings to ensure optimal sequence accuracy.

### 2.3. Sequence and Phylogenetic Analysis

ORFfinder with a minimal ORF length of 150 nt and genetic code 1 (https://www.ncbi.nlm.nih.gov/orffinder/, accessed on 11 January 2026) was used to predict the open reading frames (ORFs). InterPro https://www.ebi.ac.uk/interpro/search/sequence-search, accessed on 11 January 2026) and the NCBI Conserved domain database-CDD v3.20 (https://www.ncbi.nlm.nih.gov/Structure/cdd/wrpsb.cgi, accessed on 11 January 2026) with an e-value threshold of 0.01 was employed to identify the functional domains and architecture of translated gene products. In addition, HHPred and HHBlits, as implemented in https://toolkit.tuebingen.mpg.de/, accessed on 11 January 2026, were used to complement the annotation of divergent predicted proteins using hidden Markov models. To determine the taxonomic position of the beny-like virus, the full-length replicase proteins were used, while the full-length polyproteins were used for the picorna-like viruses, and the RdRp protein was used for the sobemo-like viruses. Phylogenetic analysis based on these mentioned proteins were carried out using MAFFT 7.526 (https://mafft.cbrc.jp/alignment/software/, accessed on 11 January 2026) with multiple amino acid (aa) sequence alignments using G-INS-i (sobemo-like) and E-INS-i (picorna-like and beny-like) as the best-fit model, respectively. The aligned aa sequences were used as input to generate phylogenetic trees through the maximum-likelihood method with the FastTree 2.1.11 tool available at http://www.microbesonline.org/fasttree/, accessed on 11 January 2026. Local support values were calculated with the Shimodaira–Hasegawa test (SH) and 1000 tree resamples. The sequences of proteins of relevant viral families related to those identified viruses were used as outgroup in the phylogenetic trees.

### 2.4. Nucleic Acid Extraction, RT-PCR Testing, and Sanger Sequencing

Psyllids were collected in yerba mate fields and total RNA was extracted from 10 pooled insects using a modified CTAB protocol (Doyle & Doyle, 1987 [19]). Reverse transcription was performed using 5 μL of the total nucleic acid extract in a 20 μL reaction mixture that contained 5× first-strand buffer (Promega, Madison, WI, USA), 1 μL 10 mM dNTP, 1 µL 250ng/ul random hexamers, and 0.8 μL M-MLV reverse transcriptase (Promega). Before the reverse transcription reaction, the RNA template was incubated at 70 °C for 5 min, then the reverse transcription mix was added. The profile used consisted of incubation at 37 °C for 60 min and reverse transcriptase deactivation at 70 °C for 10 min. All polymerase chain reactions (PCR) were accomplished by Pegasus Taq polymerase (Productos Bio-Lógicos SA, Quilmes, Argentina) in a 25 μL reaction mixture that contained 2.5 μL 10× Buffer, 1 μL 10 mM dNTP, 1 μL 10 μM of each forward primer and reverse primer, 2.4 units of Pegasus Taq Polymerase, 15.9 μL free RNAse/DNAse water and 2 μL cDNA template. The PCR profile consisted of denaturing at 94 °C for 5 min, and 40 cycles of 94 °C for 40 s, 53–60 °C for 45 s (depending on the melting temperature of primers used), 72 °C for 1 min, followed by a final extension for 10 min at 72 °C. Testing for the presence of each one of the five identified viruses was conducted using those primers listed in the Supplementary Table S1, which were designed according to the assembled sequences for each one of the five viruses. For Sanger sequencing, PCR fragments were purified with Gel/PCR DNA Fragments Extraction Kit (Geneaid Biotech Ltd., New Taipei City, Taiwan), and submitted for sequencing to Macrogen, Inc.

## 3. Results and Discussion

### 3.1. Viruses Associated with G. spegazziniana

Insects represent the most numerous and widespread group of animals on our planet [19]. The widespread use of HTS have revealed that they harbor a highly complex and diverse associated virome [16,20,21,22,23,24]. Despite these technological advances, our current knowledge still captures only a tiny fraction of the full diversity present within insect-associated viromes [25,26]. The viromes of just four psyllid species (*Bactericera cockerelli*, *Diaphorina citri*; *Leuronota fagarae* and *Trioza erytreae*) were characterized so far and several viruses were identified [27,28,29,30,31,32,33,34,35]. Moreover, the data mining of picornavirales from publicly available insect RNA-seqs datasets resulted in the identification of two novel viruses associated with two distinct psyllid species [36], highlighting the potential richness of the virome associated with psyllids. To date, around 4000 psyllid species have been described and classified [1], each exhibiting distinct life histories and host exploitation strategies [2]. This highlights how little is currently known about viruses associated with psyllids, with existing data offering an incomplete and likely misleading picture of their true viral diversity; therefore, it is tempting to speculate that the viromes of important agronomic psyllid pests, such as the yerba mate psyllid, remain largely uncharacterized. Thus, in this research, we characterized the virome of *G. spegazziniana*, which resulted in the identification of five novel highly divergent RNA viruses, including a beny-like virus, a picorna-like virus, and three sobemo-like viruses, which were tentatively named Gyropsylla spegazziniana beny-like virus 1 (GSBlV1), Gyropsylla spegazziniana picorna-like virus 1 (GSPlV1), and Gyropsylla spegazziniana sobemo-like virus 1 (GSSlV1), Gyropsylla spegazziniana sobemo-like virus 2 (GSSlV2), and Gyropsylla spegazziniana sobemo-like virus 3 (GSSlV3). The first exploration of the virome associated with *G. spegazziniana* highlights how rich and diverse is the virome associated with this highly important yerba mate pest. We assembled the full-length coding region for all viruses, and we also detected all these viruses in field-collected psyllids, suggesting that these viruses are circulating in *G. spegazziniana* populations. Uncovering the viruses associated with this major pest is a key step toward harnessing them as precise, species-specific agents for managing insect populations through biological control strategies.

### 3.2. Molecular and Phylogenetic Characterization of the Newly Identified Yerba Mate Psyllid Beny-like Virus

**Figure 1:**
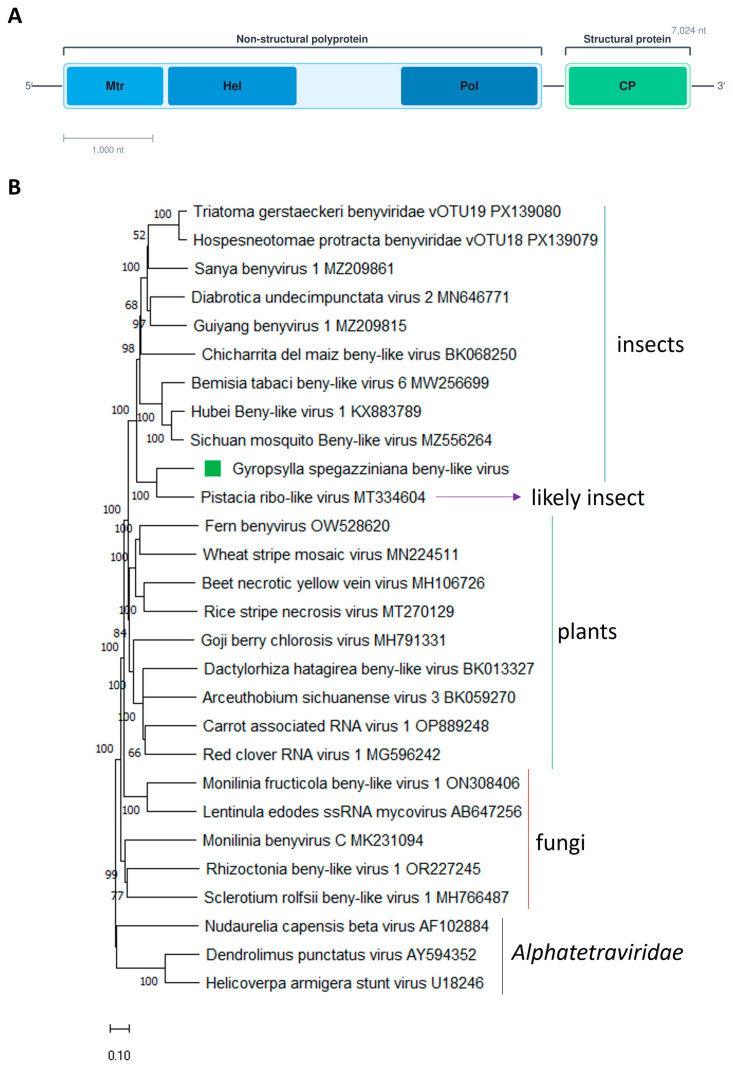
(**A**) Schematic representation of the genome organization of Gyropsylla spegazziniana beny-like Virus 1. Mtr, methyltransferase; Hel, helicase; Pol, RNA polymerase; CP, capsid protein. (**B**) Maximum likelihood phylogenetic trees reconstructed using the replicase protein sequence of Gyropsylla spegazziniana beny-like virus 1 and of representative beny-like viruses. Bootstrap values above 50% are shown (1000 replicates). Gyropsylla spegazziniana beny-like 1 virus is indicated with a green square. The scale bar shows the substitution per site.

The *Benyviridae* family comprises viruses characterized by a positive-sense, single-stranded RNA genome and currently includes a single officially recognized genus, *Benyvirus* [37]. Members of this family are primarily known to infect plants and typically possess multipartite genomes [37,38]. However, metagenomic studies as well as the metatrancriptomic analysis of public data deposited in the NCBI have uncovered numerous sequences related to benyviruses. Many of these sequences appear to represent previously unclassified members of the *Benyviridae* lineage and suggest a broader host range that extends beyond plants to include organisms such as insects and fungi [39,40,41,42,43,44,45]. Notably, viruses associated with insect hosts have a monopartite genome organization, in contrast to the segmented genomes observed in their plant-infecting relatives [39]. In this study, we identified a novel beny-like virus that was named Gyropsylla spegazziniana beny-like virus 1 (GSBlV1). The GSBlV1 genome (GenBank accession PZ145640) consists of a 7024-nucleotide positive-sense, single-stranded RNA. It contains two open reading frames (ORFs): the first encodes a large non-structural polyprotein of 1945 aa, while the second encodes a smaller protein of 274 aa that is presumed to be structural (Figure 1A; Table 1). This arrangement is consistent with the genomic architecture described for other beny-like viruses associated with insects [39,40,41,42,43,44,45], as well as for Pistacia ribo-like virus (PiRbLV; MT334604; Mohammadi et al., unpublished). Comparative analysis using BlastP indicated that the GSBlV1 non-structural polyprotein is most similar to that of PiRbLV, sharing 57.92% aa identity (Table 1). This level of divergence supports the classification of GSBlV1 as a distinct viral member that is distantly related to other insect-associated viruses currently deposited in sequence databases. Examination of the polyprotein sequence revealed the presence of three conserved functional domains: an alphavirus-like methyltransferase (MT) domain located between aa residues 60 and 282, a Viral_helicase1 (Hel) spanning aa residues 591 to 757, and the ps-ssRNAv_RdRp-like (pol) domain between aa residues 1584 and 1881 (Figure 1A). Comparable domain arrangements have also been reported in related beny-like virus polyproteins [39,40,42,43,44]. In contrast to plant-infecting members, which often encode a papain-like protease [38,44], no such protease domain was detected in the replicase of GSBlV1 or in those of other insect-associated beny-like viruses [39,40,42,43]. Regarding the second ORF, the structural encoded protein showed its closest similarity to the corresponding protein of PiRbLV, with 55.35% identity (Table 1). This protein is likely to function as a coat protein, as evidenced by the identification of a conserved TMV_coat superfamily domain spanning aa 123 to 274. Similar coat-protein-related domains have also been identified in the structural proteins of other insect-associated beny-like viruses [39,43].

Phylogenetic reconstruction based on the aa sequence of the replicase (non-structural polyprotein) grouped GSBlV1 within a cluster composed of beny-like viruses linked to insects, along with a single virus reported from *Pistacia*. Within this lineage, GSBlV1 showed the closest relationship to PiRbLV, forming a distinct subcluster with this virus. Thus, it is highly likely that PiRbLV may not genuinely infect *Pistacia*, but instead could originate from an unidentified insect whose RNA was co-extracted with the plant RNA. The clade containing the insect-associated beny-like viruses, as well as PiRbLV, was clearly separated from those lineages comprising viruses associated with fungi and plants (Figure 1B). In light of these distinct evolutionary histories of those viruses according to their associated hosts (insects, fungi or plants), our findings are consistent with the proposal by Debat et al. [39] to establish a new genus within the family *Benyviridae*, tentatively designated “Insebenyvirus,” to encompass these insect-related viruses as well as PiRbLV. Furthermore, considering both the phylogenetic relationships and the degree of sequence divergence observed among these viruses, we propose an aa identity threshold of 80% in the replicase or/and CP either the replicase or the coat protein as a demarcation criterion for distinguishing species within this putative genus.

To our knowledge, GSBlV1 is the first reported beny-like virus associated with psyllids.

### 3.3. Molecular and Phylogenetic Characterization of the Newly Identified Yerba Mate Psyllid Picorna-like Virus

The viral order *Picornavirales* includes nine recognized families, with numerous representatives known to infect arthropod hosts [46]. Members of this order possess single-stranded RNA genomes of positive polarity [47]. Although all picornavirids share the feature of polyadenylated RNA, their genomic architectures vary considerably; genomes may be either monopartite or bipartite and can encode between one and five open reading frames [46]. Here, we identified a novel picorna-like virus, which we named *Gyropsylla spegazziniana* picorna-like virus 1 (GSPlV1) (GenBank accession no. PZ145639).

**Figure 2:**
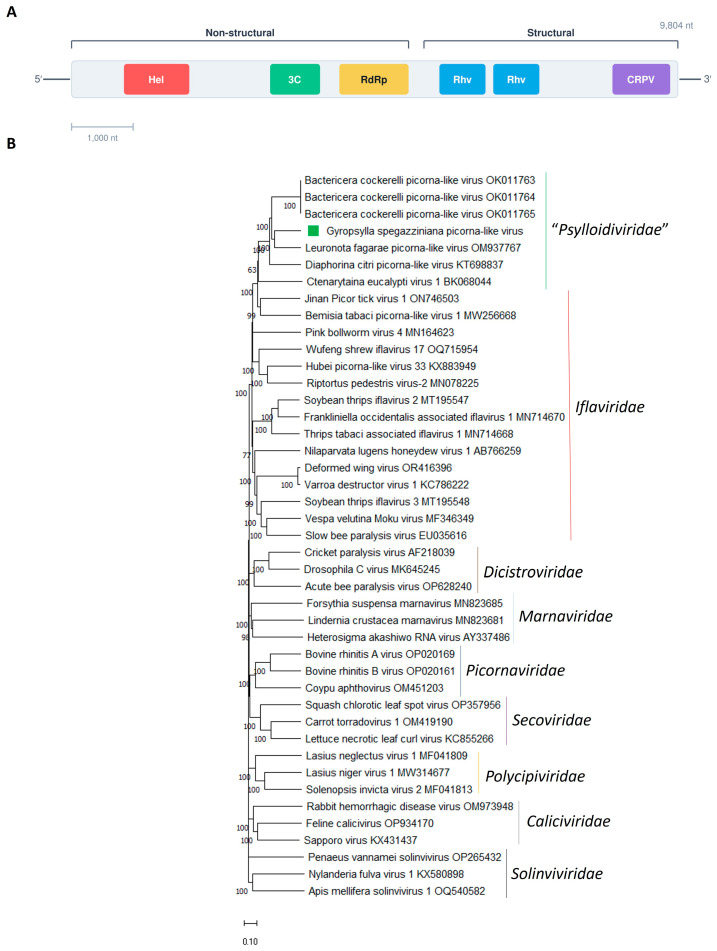
(**A**) Schematic representation of the genome organization of Gyropsylla spegazziniana picorna-like virus 1. Hel, helicase; 3C, 3C-like protease; RdRp, RNA-directed RNA polymerase; Rhv, rhinovirus-like capsid protein; CRPV, cricket paralysis virus-like capsid. (**B**) Maximum likelihood phylogenetic trees reconstructed using the polyprotein protein sequence of Gyropsylla spegazziniana picorna-like virus 1 and of representative picornavirids. Bootstrap values above 50% are shown (1000 replicates). Gyropsylla spegazziniana picorna-like virus 1 is indicated with a green square. The scale bar shows the substitution per site.

GSPlV1 has a positive-sense 9804 nt RNA genome encoding a single 2922 aa polyprotein (Figure 2A, Table 1).

Comparative BlastP analysis indicated that the GSPlV1 polyprotein is most closely related to that of the psyllid-associated *Leuronota fagarae* picorna-like virus (LfPLV), sharing 57.31% aa identity (Table 1). The genome layout of GSPlV1 is similar to that one described for other picorna-like viruses linked to psyllids [28,30,34,36], in which those non-structural domains, which are involved in the replication, are positioned toward the N-terminus, whereas those structural domains are found in the C-terminal portion of the polyprotein (Figure 2B). Within the N-terminal segment of the GSPlV1 polyprotein, the detected conserved domains were an RNA helicase (RNA_Helicase, aa 666–840), a Picornavirales 3C/3Clike protease (3C-Pro, aa 1241–1448), and a RNA-directed RNA polymerase (RdRp, aa 1477–1985) (Figure 2A). In contrast, the C-terminal region contains conserved domains associated with virion structure, specifically two rhinovirus-like capsid regions (Rhv-like, aa 2003–2244 and aa 2267–2512) as well as a cricket paralysis virus-like capsid domain (CRPV_capsid superfamily, aa 2685–2914) (Figure 2A).

Iflaviruses are known to initiate translation through a type IV internal ribosome entry site (IRES), similar to those described in dicistroviruses, located within the 5′ untranslated region (UTR) [48]. Analysis of the GSPlV1 5′UTR secondary structure using the RNAfold web server revealed a densely folded, multi-domain configuration that includes several conserved structural elements typically associated with IRES activity and essential for ribosome binding [30]. These features indicate that the 5′UTR of GSPlV1 likely serves as an IRES. Consistent with observations reported for Diaphorina citri picorna-like virus [30], and LfPLV [34], the predicted IRES element in GSPlV1 differs from the canonical type IV IRES characteristic of iflaviruses. This divergence lends support to previous studies which suggested that such atypical structural arrangements may correspond to a distinct or previously unrecognized class of IRES elements [30,34].

The conserved domains found in the GSPlV1 polyprotein are typical of picornaviruses [30,46] supporting the placement of this newly identified virus within the order *Picornavirales*. Phylogenetic analysis using the complete polyprotein sequence places GSPlV1 within a cluster composed of viruses associated with psyllids (Figure 2B). This clustering is consistent with the hypothesis proposed by Du et al. [30], suggesting that viruses infecting psyllids likely originated from a relatively recent common ancestor characterized by an inverted genome organization relative to that of members of the family *Iflaviridae*. Although GSPlV1 and related psyllid-associated viruses branch near the *Iflaviridae*, they consistently form a separate and well-supported clade (Figure 2B). In addition, GSPlV1 exhibits multiple genomic features shared with other psyllid-associated viruses [28,30,34,36] that clearly distinguish this group from iflaviruses. These include: (i) a reversed genome layout, in which non-structural proteins are encoded at the N-terminal portion of the polyprotein and structural proteins at the C-terminal end, opposite to the arrangement observed in all characterized iflaviruses; (ii) a 5′UTR containing an IRES element with a structural configuration that deviates from the canonical type described in iflaviruses; and (iii) a relatively low level of aa similarity, with the polyprotein sharing less than 40% identity between these viruses and those recognized iflaviruses. Taken together, these features support the placement of GSPlV1 within the recently proposed family “Psylloidiviridae,” specifically in the tentative genus “Psylloidivirus” [30]. The discovery of GSPlV1, along with additional newly described psyllid-associated viruses [36], provides further evidence in favor of formal recognition of this proposed taxa by the ICTV.

A number of picornaviruses have been shown to negatively impact their insect hosts, causing effects such as physical abnormalities, altered behavior, and in some cases, mortality [21]. Therefore, it will be crucial to investigate whether this newly identified picorna-like virus associated with yerba mate psyllids exhibits pathogenic effects on its insect host. Such studies could help determine its potential utility as a biological control agent for managing psyllid populations in yerba mate crops.

### 3.4. Molecular and Phylogenetic Characterization of the Newly Identified Yerba Mate Psyllid Sobemo-like Virus

Solemoviridae represents a relatively newly defined family of positive-sense RNA viruses, with all currently characterized members linked to plant hosts [49]. In general, viruses within this family possess a single, non-segmented genome that encodes between four and ten ORFs. These ORFs encode key functional proteins, including an RNA silencing suppressor, a polyprotein containing conserved domains such as a serine protease and an RNA-dependent RNA polymerase, and a capsid protein [49]. However, more recent studies have reported the existence of sobemo-like viruses exhibiting a bipartite genome organization in arthropods, including crustaceans [50], myriapods [16], and multiple insect species [29,33,51,52,53,54,55].

In this study, three sobemo-like viruses were identified and named as Gyropsylla spegazziniana sobemo-like virus 1 (GSSlV1) (GenBank accession number PZ145641-PZ145642), Gyropsylla spegazziniana sobemo-like virus 2 (GSSlV2) (GenBank accession number PZ145643-PZ145644) and Gyropsylla spegazziniana sobemo-like virus 3 (GSSlV3) (GenBank accession number PZ145645-PZ145646) (Table 1), which increases the group of sobemo-like viruses with bi-segmented genomes associated with non-plant hosts. A sobemo-like virus was previously identified in the psyllids *Bactericera cockerelli* [29] and *Trioza eritreae* [33]. Thus, *G. spegazziniana* is likely the third psyllid host where sobemo-like viruses associated with these insects have been identified.

**Figure 3:**
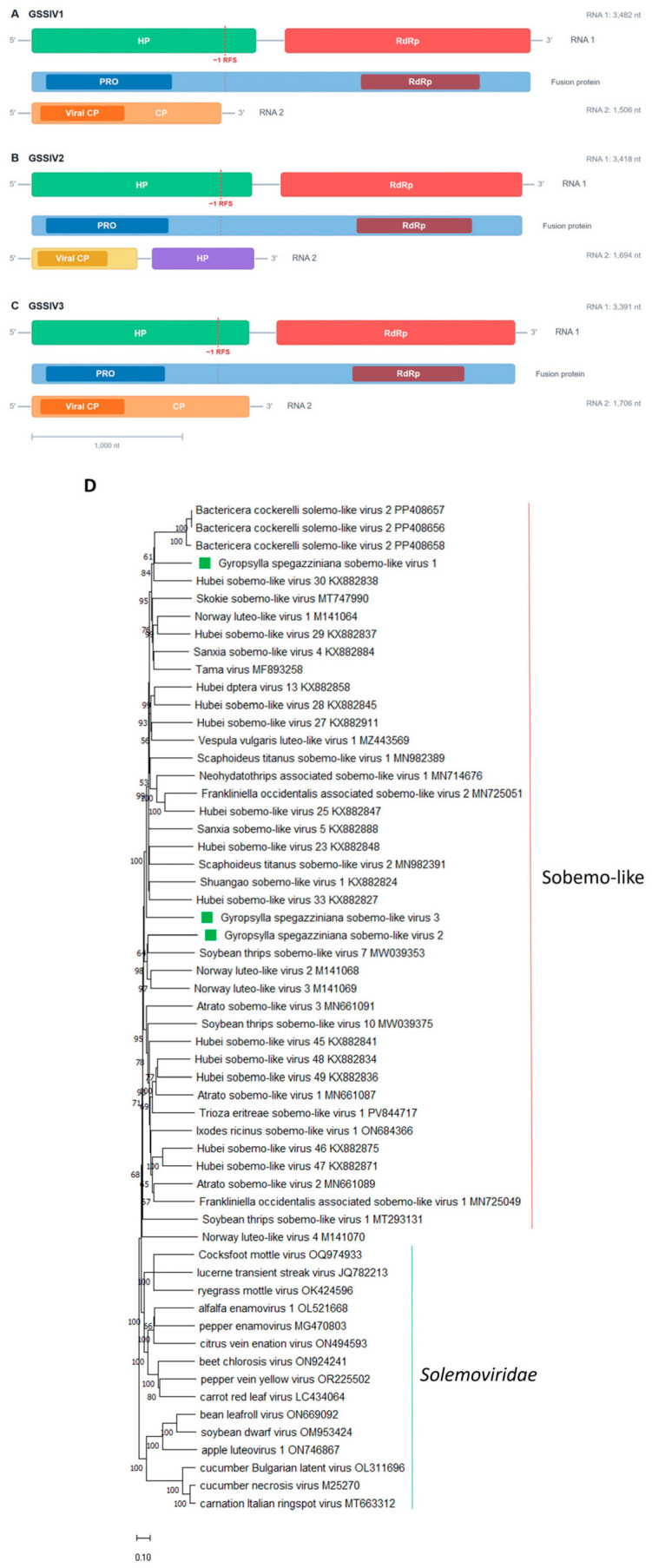
(**A**) Schematic representation of the genome organization of Gyropsylla spegazziniana sobemo-like virus 1. (**B**) Schematic representation of the genome organization of Gyropsylla spegazziniana sobemo-like virus 2. (**C**) Schematic representation of the genome organization of and Gyropsylla spegazziniana sobemo-like virus 3. Pro, Trypsin-like serin protease; RdRp, RNA-directed RNA polymerase; Viral CP, viral_coat. (**D**) Maximum likelihood phylogenetic trees reconstructed using the RdRp protein sequence of Gyropsylla spegazziniana sobemo-like virus 1, Gyropsylla spegazziniana sobemo-like virus 2 and Gyropsylla spegazziniana sobemo-like virus 3 and of representative sobemo-like viruses and plant-associated solemovirids. Bootstrap values above 50% are shown (1000 replicates). Gyropsylla spegazziniana sobemo-like virus 1, Gyropsylla spegazziniana sobemo-like virus 2 and Gyropsylla spegazziniana sobemo-like virus 3 are indicated with green squares. The scale bar shows the substitution per site.

The genomes of GSSlV1, GSSlV2, and GSSlV3 are organized into two distinct segments of single-stranded RNA with positive polarity (Figure 3A–C and Table 1). For each virus, the length of the first RNA segment is 3482 nt, 3418 nt, and 3391 nt, respectively. In contrast, the second segment is shorter, with lengths of 1506 nt, 1694 nt, and 1706 nt for GSSlV1, GSSlV2, and GSSlV3, respectively (Table 1).

Segment 1 of GSSlV1, GSSlV2, and GSSlV3 contains two predicted ORFs, designated HP and RdRp. The HP ORF encodes a putative hypothetical protein (HP), whereas the second ORF encodes the RNA-dependent RNA polymerase (RdRp), which is expressed as a fusion polyprotein via a −1 ribosomal frameshifting event (Figure 3A–C). The HPs differ in length among the three viruses: GSSlV1 encodes a protein of 713 aa, GSSlV2 encodes a 672 aa HP, and GSSlV3 encodes a 593 aa HP. Sequence comparisons using BlastP revealed that the GSSlV1 HP shares its highest similarity (36.65% identity) with Bactericera cockerelli solemo-like virus 2. In contrast, the closest match for GSSlV2 HP is Culex inatomii luteo-like virus (28.42% identity), while GSSlV3 HP is most similar to Moscow lipoptena solemo-like virus (29.54% identity). Analysis of conserved domains identified a trypsin-like serine protease (PRO) domain within all three HPs, located at aa positions 241–404 in GSSlV1, 185–344 in GSSlV2, and 213–363 in GSSlV3 (Figure 3A–C). The RdRp proteins also vary in size, consisting of 396 aa in GSSlV1, 345 aa in GSSlV2, and 375 aa in GSSlV3. BlastP analysis showed that the GSSlV1 RdRp is most closely related to Bactericera cockerelli solemo-like virus 2, with 59.39% identity. For GSSlV2, the highest similarity (41.03%) was observed with Tartas insect-associated virus, whereas GSSlV3 RdRp is most similar to Sanxia sobemo-like virus 4, sharing 46.53% identity. In all three cases, a conserved viral RNA-dependent RNA polymerases (RdRp) domain was detected, spanning aa residues 59–303 in GSSlV1, 49–248 in GSSlV2, and 37–257 in GSSlV3 (Figure 3A–C). The arrangement of genes within segment 1 of GSSlV1, GSSlV2, and GSSlV3 closely mirrors that observed in other arthropod-associated sobemo-like viruses [29,33,51,52,53,54,55]. This genomic organization resembles the 5′-proximal portion of genomes described for members of the *Solemoviridae* family, as the first ORF encodes a polyprotein that includes a serine protease domain, whereas the RdRp is synthesized from the downstream ORF through a −1 programmed ribosomal frameshifting (−1 PRF) mechanism [55]. However, unlike plant-infecting solemovirids, these viruses lack the ORF responsible for encoding a suppressor of RNA silencing, consistent with their non-plant hosts [55].

Segment 2 of GSSlV1 and GSSlV3 each contains a single ORF encoding a capsid protein (CP) (Figure 3A,C), with lengths of 416 and 435 aa, respectively. Comparative BlastP analysis indicates that the CP of GSSlV1 shares its highest similarity (36.41% identity) with Scaphoideus titanus sobemo-like virus 1, whereas the CP of GSSlV3 is most closely related to Neohydatothrips-associated sobemo-like virus 1, exhibiting 35.44% identity. In contrast, segment 2 of GSSlV2 displays a more complex organization, comprising two ORFs (Figure 3B). These ORFs encode a capsid protein of 219 aa and an additional hypothetical protein (HP) of 248 aa. The CP of GSSlV2 shows its closest match to Chihuahua culicoides solemo-like virus 1, with 33.62% identity, while the HP exhibits the highest similarity (30.19% identity) to Trioza erytreae sobemo-like virus. Domain analysis revealed the presence of a conserved viral coat protein (Viral CP) domain in the CPs of all three viruses. This domain spans aa residues 59–229 in GSSlV1, 65–234 in GSSlV2, and 21–216 in GSSlV3 (Figure 3A–C). In contrast, no recognizable conserved domains were detected in the hypothetical protein encoded by segment 2 of GSSlV2. Interestingly, any segment 2 was reported for the psyllid-associated Bactericera cockerelli solemo-like virus 2 [29]. Thus, it is tempting to speculate that authors did not search that segment in their data. Therefore, we analyzed the SRA28090463 associated with the bioproject PRJNA1080349 [29] which resulted in the identification of the putative segment 2 of the Bactericera cockerelli solemo-like virus 2 with 1617 nt, that encodes a 415aa CP, which shares a 47.8% identity to the one encoded by GSSlV1. These results strengthen the idea that sobemo-like viruses associated with arthropods consistently have bipartite genomes, even though earlier reports frequently described only a single genomic segment for many of these viruses. In addition, the capsid proteins of GSSlV1, GSSlV2, and GSSlV3, as well as from other arthropod-linked sobemo-like viruses, exhibit detectable sequence similarity to those found in noda-like and permutotetra-like viruses. This pattern is consistent with the fact that, in those viral groups, CPs are often encoded by subgenomic RNAs or by separate genomic segments. Consequently, the observed similarities may be explained by horizontal gene transfer events, potentially arising from genome segment mispackaging during co-infection of a shared host organism [55].

Phylogenetic reconstruction using RdRp aa sequences positioned GSSlV1, GSSlV2, and GSSlV3 within a broader lineage of unclassified solemovirid-related viruses detected in diverse arthropod hosts. Within this group, GSSlV1 grouped closely with Bactericera cockerelli solemo-like virus 2 and Hubei sobemo-like virus 30. GSSlV2 clustered together with soybean thrips sobemo-like virus 7, whereas GSSlV3 occupied a distinct, well-supported monophyletic branch (Figure 3D).

GSSlV1, GSSlV2 and GSSlV3 share very low identity in their encoded proteins and are located in distinct clades, indicating that these three viruses likely have distinct evolutionary trajectories.

Considering both their evolutionary relationships and genome architecture, the group of solemovirid-related viruses infecting non-plant hosts, particularly arthropods, appears sufficiently distinct to justify the creation of a new viral family within the order *Sobelivirales*, with additional genera to be further assigned. Moreover, the unique genomic features observed in these non-plant-associated viruses highlight the need for further investigation into their replication strategies and the evolutionary processes that gave origin to this lineage.

### 3.5. GSBlV1, GSPlV1, GSSlV1, GSSlV2 and GSSlV3 Can Be Found in Field-Collected Psyllids

Psyllids were field-collected in yerba mate crops and tested for the presence of the five identified viruses using RT-PCR with those primers listed in Supplementary Table S1. All five viruses were successfully amplified from the field-collected samples; for GSSlV1, GSSlV2 and GSSlV3 their RNA 1 was targeted, showing the expected size of the PCR bands when tested with the appropriate primers for each virus (Supplementary Figure S1). These amplified fragments were submitted for Sanger sequencing which resulted in nucleotide sequences more than 99% identical to those derived from the HTS assembled sequences for the five viruses. The primers developed in this study provide a useful tool for future investigations aimed at evaluating the prevalence of each of the five viruses detected in *G. spegazziniana* populations from different geographic locations. Such analyses would also enable a better understanding of viral dynamics within yerba mate psyllid populations, helping to clarify whether these viruses contribute to the natural regulation of psyllid abundance in yerba mate plantations. Comparable research efforts in other psyllid species are already underway [29,56], and these studies will offer valuable insights into infection patterns and the potential role of viruses as biological control agents in psyllid populations.

## 4. Concluding Remarks

Through transcriptomic analysis of the yerba mate psyllid, five novel viruses were discovered, expanding current understanding of the viral diversity associated with this economically significant pest and providing new insights into the evolutionary relationships among psyllid-infecting viruses. These findings further highlight the power of metagenomic and metatranscriptomic approaches to reveal a much broader spectrum of viral diversity within psyllid species. The detection of all five viruses in field-derived insect samples indicates that they are likely widely distributed across natural populations of *G. spegazziniana*. This work also opens new avenues for investigating the biological roles of these viruses within their host. In particular, studies focusing on virus–host interactions and functional characterization will be essential to determine how these viruses affect psyllid physiology, behavior, and responses to environmental conditions. Gaining insights into these virus–host interactions could pave the way for innovative and environmentally sustainable pest management strategies, potentially by targeting specific viral mechanisms or altering virus-mediated effects on the biology of the yerba mate psyllid. In addition, a comprehensive understanding of the virome is essential for the design of virus-based bio-insecticides. Characterizing the viral community associated with *G. spegazziniana* could therefore provide a foundation for developing new virocontrol strategies aimed at sustainably reducing populations of this agriculturally important pest.

## Figures and Tables

**Table 1:** Summary of the viruses identified.

Virus Name/Abbreviation	Accession Number	Length (nt)/Coverage	Protein ID/Length (aa)	Highest Scoring Virus- Protein/*E*-Value/Query Coverage%/Identity% (Blast P)
Gyropsylla spegazziniana beny-like virus 1/ GSBlV1	PZ145640	7024/118.76X	replicase/1945 coat protein/274	PiRbLV-replicase/0.0/100/57.92 PiRbLV-coat protein/2e-62/74/55.35
Gyropsylla spegazziniana picorna-like virus 1/GSPlV1	PZ145639	9804/2618.39X	polyprotein/2922	LfPLV-polyprotein/0.0/100/57.31
Gyropsylla spegazziniana sobemo-like virus 1/ GSSlV1	PZ145641 PZ145642	RNA 1 3482/4863.81X RNA 2 1506/4479.59X	fusion protein/1115 coat protein/416	BcSLV2-fusion protein/0.0/98/58.22 SctSLV1-coat protein/9e-65/85/36.41
Gyropsylla spegazziniana sobemo-like virus 2/ GSSlV2	PZ145643 PZ145644	RNA 1 3418/2603.82X RNA 2 1694/2381.21X	fusion protein/1064 coat protein/219 hypothetical protein/248	StSLV7- fusion protein/0.0/95/38.33 CcSLV1-coat protein/1e-27/100/33.62 TeSLV-hypotetical protein/1e-24/85/30.19
Gyropsylla spegazziniana sobemo-like virus 3/ GSSlV3	PZ145645 PZ145646	RNA1 3391/7469.62X RNA2 1706/4479.59X	fusion protein/1047 coat protein/435	SctSLV2- fusion protein/0.0/96/43.88 NTaSLV1-coat protein/1e48/75/35.44

## Data Availability

The sequences were deposited in GenBank with accession numbers PZ145639-PZ145646.

## Supplementary Materials

The following supporting information can be downloaded at: https://www.mdpi.com/article/10.3390/pathogens15060620/s1, Figure S1: Testing the five viruses by endpoint RT-PCR in a bulked sample of ten field-collected yerba mate psyllids; Table S1: List of primer pairs used in this study.
