# Supplementary material for: Exploring the Virome of Gyropsylla spegazziniana: A Major Yerba Mate Pest
Source: Pathogens. 2026 Jun 9;15(6):620. doi: 10.3390/pathogens15060620 (PMC13306095; doi:10.3390/pathogens15060620)
Supplement: Supplementary file 1 [file pathogens-15-00620-s001.zip › pathogens-4275147-supplementary.pdf]

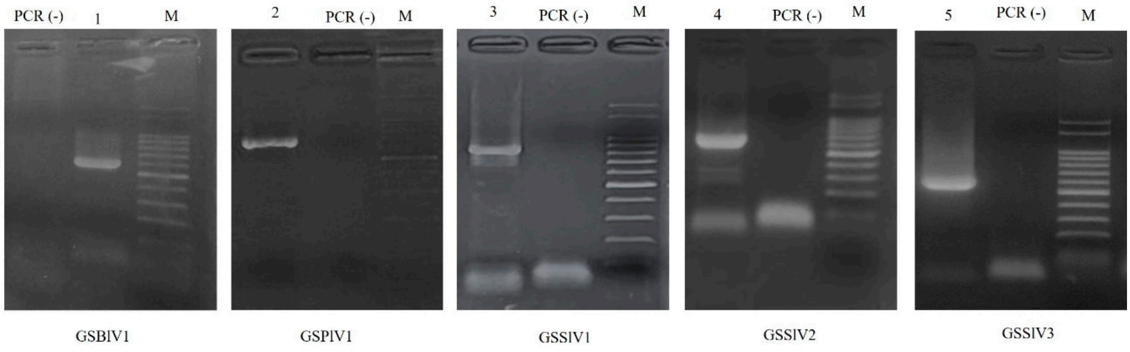

Supplementary Figure S1. Testing the five viruses by endpoint RT-PCR in a bulked sample of ten field-collected yerba mate psyllids; M: molecular marker, 100 bp DNA ladder (PB-L Productos Bio-Lógicos, Argentina (100, 200, 300, 400, 500, 600, 700, 800, 900, 1000, 1500 and 2000 pb)). PCR (-): PCR negative control. 1) Gyropsylla spegazziniana beny-like virus 1 (GSBlV1) (703pb), 2) Gyropsylla spegazziniana picorna-like virus 1 (GSPlV1) (566pb), 3) Gyropsylla spegazziniana sobemo-like virus 1 (GSSlV1) (890pb), 4) Gyropsylla spegazziniana sobemo-like virus 2 (GSSlV2) (759pb), and 5) Gyropsylla spegazziniana sobemo-like virus 3 (GSSlV3) (793pb).

Supplementary Table S1. List of primer pairs used in this study. The sequences for all primer pairs used for RT-PCR testing of each virus (GSB1V1, GSP1V1, GSS1V1, GSS1V2 and GSS1V3) are given in the table, with start and end positions based on the corresponding contig assembled from HTS data as escribed in main text, Tm for each primer as calculated using Primer-BLAST (NCBI) with default parameters, and length of predicted PCR product.

Primer name	Start	End	Sequence	Tm (°C)	Length (bp)
**GSB1V1_F**	3093	3112	TGTCGACGGAGACACAACAG	59.97	703
**GSB1V1_R**	3776	3795	CTCGGACGATGTACCGGAAG	59.97	
**GSP1V1_F**	1618	1637	CGGCAGCGTCAGAAATAACG	60.03	566
**GSP1V1_R**	2164	2183	ATGATCCGGTTTGCCACCAT	60.0	
**GSS1V1_F**	1157	1176	CGGAATGAGTGGAGCACCTT	60.04	890
**GSS1V1_R**	2027	2046	TCTTCCGAGAGCTCTTCCCT	59.67	
**GSS1V2_F**	357	376	CTTGTGTCGGTTGGCAGTTG	53.83	759
**GSS1V2_R**	1096	1115	CACGTCCAGAATCCCCACAA	53.83	
**GSS1V3_F**	1765	1784	AACTGGGCAGGGAGCATAAC	59.97	793
**GSS1V3_R**	2538	2557	CGCCTCATTTTGCCAACCAA	59.97	
